# Stability of African swine fever virus genome under different environmental conditions

**DOI:** 10.14202/vetworld.2023.2374-2381

**Published:** 2023-11-27

**Authors:** Wei Zheng, Jiahui Xi, Yin Zi, Jinling Wang, Yue Chi, Min Chen, Qingjian Zou, Chengcheng Tang, Xiaoqing Zhou

**Affiliations:** Guangdong Provincial Key Laboratory of Large Animal Models for Biomedicine, South China Institute of Large Animal Models for Biomedicine, School of Biotechnology and Health Science, Wuyi University, Jiangmen, 529000, China

**Keywords:** African swine fever, enzyme-linked immunosorbent assay, quantitative polymerase chain reaction, standard disinfection methods, virus stability

## Abstract

**Background and Aim:**

African swine fever (ASF), a globally transmitted viral disease caused by ASF virus (ASFV), can severely damage the global trade economy. Laboratory diagnostic methods, including pathogen and serological detection techniques, are currently used to monitor and control ASF. Because the large double-stranded DNA genome of the mature virus particle is wrapped in a membrane, the stability of ASFV and its genome is maintained in most natural environments. This study aimed to investigate the stability of ASFV under different environmental conditions from both genomic and antibody perspectives, and to provide a theoretical basis for the prevention and elimination of ASFV.

**Materials and Methods:**

In this study, we used quantitative real-time polymerase chain reaction for pathogen assays and enzyme-linked immunosorbent assay for serological assays to examine the stability of the ASFV genome and antibody, respectively, under different environmental conditions.

**Results:**

The stability of the ASFV genome and antibody under high-temperature conditions depended on the treatment time. In the pH test, the ASFV genome and antibody remained stable in both acidic and alkaline environments. Disinfection tests revealed that the ASFV genome and antibody were susceptible to standard disinfection methods.

**Conclusion:**

Collectively, the results demonstrated that the ASFV genome is highly stable in favorable environments but are also susceptible to standard disinfection methods. This study focuses on the stability of the ASFV genome under different conditions and provides various standard disinfection methods for the prevention and control of ASF.

## Introduction

African swine fever (ASF) is a highly lethal contagious disease caused by the ASF virus (ASFV) [1]. In swine, the disease has a mortality rate of up to 100% [2], posing a huge threat to the global swine industry and causing incalculable economic losses [3]. Due to its multiple modes of transmission, more than 50 countries and regions have been affected by ASFV, and it is showing a global epidemic trend [4]. Therefore, the World Organization for Animal Health (OIE) has classified ASF as a legally reportable animal disease.

**Figure-1:**
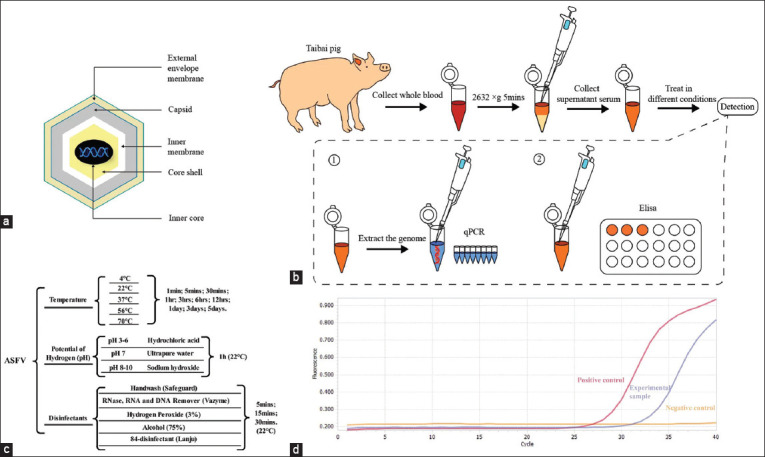
Stability detection of African swine fever virus (ASFV) genome and antibody under different environmental conditions. (a) Illustration of the ASFV particle. African swine fever virus consists of five layers: The external envelope membrane, the capsid, the inner membrane, the core shell, and the inner core. (b) Experimental procedure. Whole-blood was collected from ASFV-infected Taibai pigs, and after centrifugation to collect the supernatant serum, samples were processed under different conditions and then tested for ASFV stability by quantitative real-time polymerase chain reaction (qPCR) and enzyme-linked immunosorbent assay (ELISA). (c) The stability was examined by qPCR and ELISA by setting different treatment times and changing the environmental conditions such as temperature, pH, and different disinfectants. (d) Specific amplification curves for the experimental group and positive control in the qPCR results.

African swine fever virus and its genome can maintain their stability in most natural environments. African swine fever virus is the only member of the Asfarviridae family [5]. Mature ASFV virus particles contain a large double-stranded DNA genome. This genome encodes various enzymes and proteins that repair and protect the DNA, thereby helping maintain genome integrity and stability [6–9]. The ASFV particle is an icosahedral multilayered structure. This large, complex DNA virus contains five layers: an external envelope membrane, a capsid, an inner membrane, a core shell, and an inner core, each containing a different type of protein (Figure-1a). Due to the complex multilayered structure of the virus particles, ASFV is extremely resistant to temperature and pH. African swine fever virus can survive and spread in different seasons, climates, and pH conditions [10]. In addition, the presence of organic matter, such as blood, serum, and uncooked meat or meat products, increases the stability and survival time of the virus [11, 12].

Given the lack of vaccine or effective treatment for ASF [11], the most direct prevention and control measures are to detect and identify infected wild or domestic pigs and immediately perform extensive disinfection, quarantine, and culling. However, the clinical signs of ASFV and common swine fever are similar. A definite diagnosis of ASF by clinical signs is not possible; therefore, laboratory testing techniques are often used for diagnostic evaluation. The most common laboratory testing techniques are pathogen assays and serologic assays [13]. Pathogen assays detect viruses directly at the antigenic or genetic level and include polymerase chain reaction (PCR), erythrocyte adsorption assay, macrophage virus isolation, direct fluorescent antibody assay, and antigen-based enzyme-linked immunosorbent assay (ELISA) [14]. Serological tests detect the virus indirectly, by detecting antibodies against the virus, and include antibody-based ELISA, indirect fluorescent antibody assay, and immunoblotting [15].

Given that ASFV can be detected on different surfaces in contaminated sites, its ability to survive in the environment means that different substrates may possess the risk of transmitting the virus [16]. Therefore, choosing a suitable disinfectant and applying it effectively, considering environmental conditions, contact time, pH, and temperature ranges, plays a crucial role in the control of this virus [14]. Viruses can be classified into two groups: enveloped and non-enveloped [17]. Because ASFV is an enveloped virus, it is very sensitive to detergents, soaps, common disinfectants, and dehydration [18–23].

To better investigate the viability of the virus and discover more effective means of its inactivation, in this study, we investigated the stability of the ASFV genome under different environmental conditions using quantitative real-time PCR (qPCR) and the stability of the ASFV antibody under different environmental conditions using ELISA (Figures-1b and c). Based on these results, we also provide various standard disinfection methods for preventing and controlling ASFV.

## Materials and Methods

### Ethical approval

No live piglets were used in this study. All the blood samples were collected from a farm. Ethical approval was not necessary for our study as per the guideline of School of Biotechnology and Health Science, Wuyi University, Guangdong, China.

### Study period and location

The study was conducted from March to August 2022. All experiments were performed at Guangdong Provincial Key Laboratory of Large Animal Models for Biomedicine, South China Institute of Large Animal Models for Biomedicine, School of Biotechnology and Health Science, Wuyi University, Guangdong, China.

### Collection and preservation of serum samples

Whole-blood samples were collected from Taibai pigs infected with ASFV. The supernatant serum was separated by centrifugation at 2632*×g* for 5 min using an Allegra X-30R centrifuge (Beckman Coulter, Brea, USA). After collecting 200 μL of each sample, the serum was stored in centrifuge tubes (Axygen, Union City, USA) at −80°C in an ultra-low temperature refrigerator (PHCbi, Shanghai, China). The collected serum samples were double-tested by qPCR and ELISA (Figure-1b).

### Genomic DNA extraction procedures

DNA was extracted and purified from serum samples using a viral genome DNA/RNA extraction kit (TIANamp Virus DNA/RNA Kit, Tiangen Biochemical Technology Co., Ltd., Beijing, China). African swine fever virus genomic DNA was stored at −20°C in a refrigerator (Panasonic, Japan) until further use.

### Design of primers

A pair of primers (forward: CCCTGAATCGGAGCATCCT; reverse: AGTTATG GGAAACCCGACCC) was designed for the *B646L* gene of ASFV (encoding coat protein p72), and all primers were synthesized by Suzhou Genewiz Biotechnology Co (Suzhou, China). African swine fever virus genomic DNA was tested by qPCR targeting the same gene region [24].

### Quantitative real-time PCR conditions

To assess the presence of ASFV in the samples at different time points and environments from a pathogen perspective [25], qPCR was performed to detect the *B646L* gene of ASFV. Premixes, which contained 0.3 μL of each primer (10 μM), 1 μL of template DNA (ASFV genomic DNA), and 5 μL of 2× SYBR Green qPCR Master Mix (Bimake Biotechnology, Inc., USA), were prepared to make a total volume of 10 μL. The following temperature profile was used: 5 min of initial denaturation at 95°C; 40 amplification cycles of 10 s at 95°C, 20 s at 60°C, and 30 s at 72°C for denaturation, annealing, and extension, respectively; and one cycle of the melting curve procedure, which included 15 s at 95°C, 60 s at 60°C, and 15 s at 95°C. Quantitative real-time PCR was performed using a Roche LC96 Fluorescent Quantitative PCR LightCycler 96 (Roche, Basel, Switzerland).

The test was considered valid if the positive control had a cycle threshold (CT) value of <30 and showed a specific amplification curve, if the negative control had no CT value, or if the negative control had a CT value of >40 and showed no specific amplification curve. Samples were considered positive for ASFV when the CT value was <38 and a specific amplification curve was present (Figure-1d).

### Antibody enzyme-linked immunoassay procedures

To assess the presence of ASFV in the samples from a serological point of view, ELISA was performed to indirectly detect ASFV antibodies. An ASFV antibody ELISA kit (ASF-Ab ELISA kit, Shanghai Yaji Biotechnology Co., Ltd., Shanghai, China) was used for the enzyme immunoassay detection of antibodies in sera. The serum samples were first equilibrated to room temperature (26°C ± 1°C) for 10 min until they were completely thawed. Approximately 50 μL of serum was added into the sample wells, followed by 100 μL of horseradish peroxidase (HRP)-labeled detection antigen. We then added 50 μL of the standards (ASF-Ab ELISA kit, Shanghai Yaji Biotechnology Co., Ltd., Shanghai, China) and HRP to the standard wells. The sealed reaction wells were incubated in a thermostat (PHCbi) at 37°C for 60 min, and the plates were washed 5 times. Approximately 50 μL of substrate A and 50 μL of substrate B were added to each well, and the reaction was terminated by adding 50 μL of termination solution after incubation at 37°C for 15 min in the thermostat. The absorbance (optical density) was measured at 450 nm using an enzyme standard (BioTek Synergy NEO_2_, Winooski, USA) within 15 min.

### Types and compositions of disinfectants

Because ASFV is an enveloped virus, it is very sensitive to detergents and common disinfectants [26]. Among the disinfectants active against ASFV, several types of commonly used disinfectants were selected: acids, alkalis, chlorine and chlorine compounds (84-disinfectant), oxidizing agents (hydrogen peroxide, 3%), alcohol compounds (alcohol, 75%), and detergents (handwash) [27–29]. Acidic and alkaline disinfectants were configured by mixing hydrochloric acid and sodium hydroxide, respectively, with ultrapure water. RNase, RNA, and DNA remover (Vazyme, Piscataway, USA), which is used in the laboratory to create an experimental environment free of RNAase and nucleic acid contamination, was also selected for testing. The main active ingredient in the handwash (Safeguard, Tianjin, China) was Octopirox (OCT, content: 0.18%–0.22%, W/W), and the main active ingredient in the 84-disinfectant (Lanju, Guangzhou, China) was NaCIO (chloride, effective chlorine content: 5%–7%).

## Results

### Stability of the ASFV genome and antibody in high-temperature conditions depends on the treatment time

**Figure-2:**
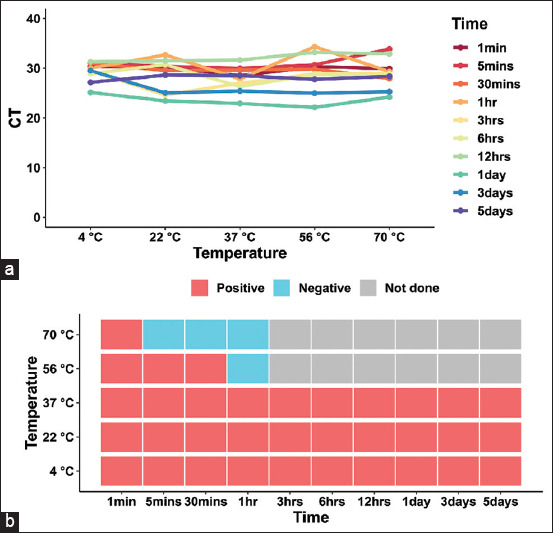
Stability of African swine fever virus (ASFV) genome and antibody at different temperatures. (a) The quantitative real-time polymerase chain reaction results of the stability of the ASFV genome at different temperatures: 4°C, 22°C, 37°C, 56°C, and 70°C. The treatment times were 1 min, 5 min, 30 min, 1 h, 3 h, 6 h, 12 h, 1 day, 3 days, and 5 days. CT = cycle threshold value. (b) The Enzyme-linked immunosorbent assay results of the stability of the ASFV antibody at various temperatures: 4°C, 22°C, 37°C, 56°C, and 70°C. The treatment times were 1 min, 5 min, 30 min, 1 h, 3 h, 6 h, 12 h, 1 day, 3 days, and 5 days.

First, we measured the stability of the ASFV genome and antibody at various temperatures (4°C, 22°C, 37°C, 56°C, and 70°C). The following treatment times were employed: 1 min, 5 min, 30 min, 1 h, 3 h, 6 h, 12 h, 1 day, 3 days, and 5 days. Although many studies have shown that ASFV exhibits prolonged stability in natural environments at 26°C ± 1°C, a temporal gradient assay in non-high-temperature environments over a short period was set up in this study [30–32]. The qPCR results showed that the treatments at different times and temperatures had only slight effects on the ASFV genome, which was detected in all samples (Figure-2a, Table-1, Supplementary data), consistent with the reported research [33]. However, antibody enzyme-linked immunoassay performed on serum demonstrated the heat sensitivity of the ASFV antibody, which was undetectable from the serum after 30 min of treatment at 56°C. When the incubation temperature was increased to 70°C, the time for ASFV antibody inactivation was reduced to 5 min (Figure-2b, Table-1 and Supplementary data). These results indicate that the stability of the ASFV genome and antibody in high-temperature conditions depends on the treatment time.

**Table-1:** Stability of ASFV genome and antibody at different temperature conditions.

Time	4°C		22°C		37°C		56°C		70°C	
				
	qPCR (CT)	ELISA	qPCR (CT)	ELISA	qPCR (CT)	ELISA	qPCR (CT)	ELISA	qPCR (CT)	ELISA
1 min	30.33	Positive	30.29	Positive	28.49	Positive	30.32	Positive	29.93	Positive
5 min	31.04	Positive	30.42	Positive	29.99	Positive	30.69	Positive	33.87	Negative
30 min	30.98	Positive	29.54	Positive	29.61	Positive	29.84	Positive	27.93	Negative
1 h	30.01	Positive	32.69	Positive	27.86	Positive	34.34	Negative	29.32	Negative
3 h	29.50	Positive	24.61	Positive	26.98	Positive	28.88	-	28.91	-
6hrs	28.91	Positive	30.73	Positive	26.31	Positive	28.74	-	28.69	-
12 h	31.28	Positive	31.51	Positive	31.64	Positive	33.20	-	32.91	-
1 day	25.13	Positive	23.44	Positive	22.92	Positive	22.13	-	24.20	-
3 days	29.50	Positive	25.04	Positive	25.43	Positive	24.97	-	25.29	-
5 days	27.16	Positive	28.62	Positive	28.52	Positive	27.77	-	28.38	-

*All experiments were done in triplicates. -=not done. ASFV=African swine fever virus, ELISA=Enzyme-linked immunosorbent assay, CT=Cycle threshold, qPCR=Quantitative real-time polymerase chain reaction

### African swine fever virus genome and antibody remain stable in both acidic and alkaline environments

**Figure-3:**
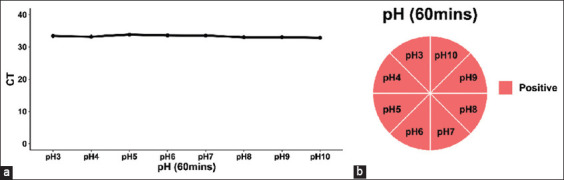
Stability of African swine fever virus (ASFV) genome and antibody at different pHs. (a) The quantitative real-time polymerase chain reaction results of the stability of the ASFV genome at different pHs: pH 3, pH 4, pH 5, pH 6, pH 7, pH 8, pH 9, and pH 10. The treatment time was 60 min. CT=Cycle threshold value. (b) The enzyme-linked immunosorbent assay results of the stability of the ASFV antibody at different pHs: pH 3, pH 4, pH 5, pH 6, pH 7, pH 8, pH 9, and pH 10. The treatment time was 60 min. All experiments were performed at room temperature (26°C ± 1°C).

African swine fever virus is considered to be highly resistant to pH, and the particles are stable in serum-free media at pH 4–10 [12]. However, acidic and alkaline disinfectants can affect ASFV activity [26]. To explore the effects of acid and alkalis on ASFV, we investigated the stability of the ASFV genome and antibody in a wide pH range (pH 3–10) at 26°C ± 1°C. A pH 3–10 solution was prepared using hydrochloric acid, sodium hydroxide, and ultrapure water. We avoided adding too much acidic or alkaline solution to the serum, as this would result in a low concentration of the virus in the mixed solution. After a gradient of volumetric mixing experiments, it was finally determined that the mixing process should be performed using an acid/alkaline solution to serum ratio of 3:10. Each pH 3–10 solution (15 μL) was mixed with serum (50 μL) and reacted at 26°C ± 1°C for 1 h. It was confirmed using pH paper that all mixtures were weakly alkaline (pH 7–8), close to the pH of the serum itself. The results of qPCR revealed that the ASFV genome was extremely stable in the tested pH range at 26°C ± 1°C (Figure-3a, Table-2 and Supplementary data). Further, experiments using ELISA revealed that the virus antibody behaved stably in both acidic (pH 3–4) and alkaline environments (pH 9–10) (Figure-3b and Table-2). Ultrapure water was used as a negative control, and the control experiments showed that the negative and positive results had no interference due to the extreme pH change in the sample. These results demonstrate that the ASFV genome and antibody remain stable in both acidic and alkaline environments, demonstrating its tenacious vitality in different environmental conditions.

**Table-2:** The stability of ASFV genome and antibody at different pH conditions.

pH (60 min)	qPCR (CT)	ELISA
3	33.38	Positive
4	33.16	Positive
5	33.80	Positive
6	33.55	Positive
7	33.48	Positive
8	33.03	Positive
9	33.03	Positive
10	32.83	Positive

*All experiments were done in triplicates. ASFV=African swine fever virus, ELISA=Enzyme-linked immunosorbent assay, CT=Cycle threshold, qPCR=Quantitative real-time polymerase chain reaction

### African swine fever virus genome and antibody are susceptible to standard disinfection methods

**Figure-4:**
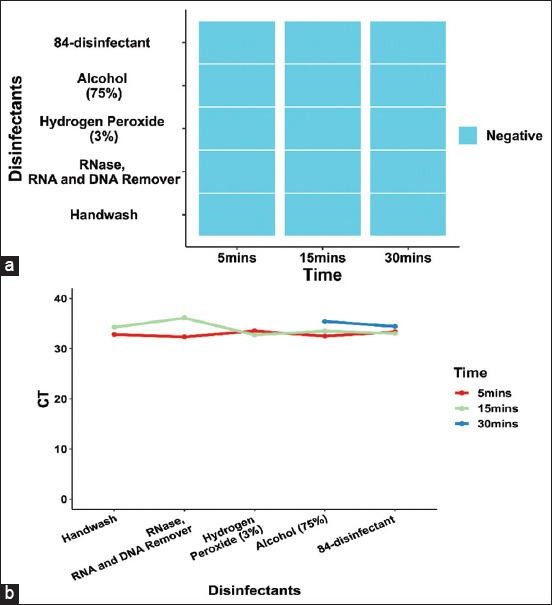
Stability of African swine fever virus (ASFV) genome and antibody after the application of various disinfectants. (a) The enzyme-linked immunosorbent assay results of stability of ASFV antibody after incubation with different disinfectants. The disinfectants included handwash, RNase, RNA, and DNA remover, hydrogen peroxide (3%), alcohol (75%), and 84-disinfectant. The treatment times were 5 min, 15 min, and 30 min. (b) The quantitative real-time polymerase chain reaction results of the stability of the ASFV genome after incubation with different disinfectants. The disinfectants included handwash, RNase, RNA, and DNA remover, hydrogen peroxide (3%), alcohol (75%), and 84-disinfectant. The treatment times were 5 min, 15 min, and 30 min. CT=Cycle threshold value. All experiments were performed at room temperature (26°C ± 1°C).

To assess the sensitivity of ASFV to disinfectants, we tested the virucidal effect of various disinfectants by adding 50 mL of ASFV-containing serum to 15 mL of various disinfectants at working concentrations. According to the ELISA results, ASFV antibodies were not detectable in any of the samples treated with disinfectant after 5 min of incubation at 26°C ± 1°C (Figure-4a, Table-3 and Supplementary data). Quantitative real-time PCR showed more detailed results, with trace amounts of ASFV genome being detected in all samples after 5 min of incubation. The samples treated with hydrogen peroxide (3%), alcohol (75%), and 84-disinfectant required more than 15 min to destroy the viral genome (Figure-4b, Table-3, Supplementary data). These results showed that the ASFV genome and antibody are susceptible to standard disinfection methods, which are essential to prevent and control the spread of ASFV.

**Table-3:** The stability of ASFV genome and antibody at different disinfectants.

Disinfectants (Working concentration)	5 min		15 min		30 min	

	qPCR (CT)	ELISA	qPCR (CT)	ELISA	qPCR (CT)	ELISA
Handwash	32.84	Negative	U	Negative	U	Negative
RNase, RNA and DNA remover	32.35	Negative	U	Negative	U	Negative
Hydrogen peroxide (3%)	33.54	Negative	32.75	Negative	U	Negative
Alcohol (75%)	32.51	Negative	33.53	Negative	U	Negative
84-disinfectant	33.37	Negative	33.05	Negative	U	Negative

*All experiments were done in triplicates. U=Undetectable, ASFV=African swine fever virus, ELISA=Enzyme-linked immunosorbent assay, CT=Cycle threshold, qPCR: Quantitative real-time polymerase chain reaction

## Discussion

The global spread of ASFV is a huge challenge worldwide. A better understanding of the stability of the ASFV genome in different environments will facilitate further prevention and control of ASF [34].

This study focused on the stability of the ASFV genome and antibody under different environmental conditions, including various temperatures, pH, and disinfectants, from both pathogenic and serological perspectives.

The qPCR results demonstrated that temperature had little effect on the ASFV genome. Despite the degradation, the sample remained positive for ASFV after 5 days of high-temperature treatment at 70°C. The most likely explanation for this is that ASVF is a DNA virus with a relatively stable genome in various environments [30, 32]. We then performed a repeat experiment on the samples from an antibody perspective. The results showed that the ASFV antibody was heat sensitive and that the virus antibody was undetectable in the serum after 30 min of treatment at 56°C. As the incubation temperature was increased to 70°C, the virus inactivation time was reduced to 5 min. A common practice among small pig farmers is to feed their pigs with table scraps or food waste that has not received adequate heat treatment, and several studies have shown that ASFV outbreaks on pig farms have been caused by feeding pigs with swill [35–37]. The OIE recommends heat inactivation of ASFV in swill by maintaining temperatures above 90°C for more than 60 min [38]. The Food and Agricultural Organization recommends heat treatment at 70°C for 30 min to inactivate ASFV [39]. Based on the results of this study, heat treatment of swill at a minimum guaranteed temperature of 56°C for 30 min is recommended. Alternatively, the temperature can be increased to shorten the treatment time.

The results of the pH investigations demonstrated that pH had a minimal effect on the ASFV genome. Similarly, the ASFV antibody was stable in both acidic and alkaline environments [40, 41]. The previous studies by Galindo *et al*. [42], Hernaez *et al*. [43], and Sanchez *et al*. [44] have suggested that ASFV infection into cells requires a low pH environment, that the disassembly of the coat and dissociation of the outer membrane processes are strongly dependent on an acidic pH, and viral particles show clear signs of disassembly and structural loss at a pH of <5.

The envelope of ASFV contains lipids, making it very sensitive to detergents, soaps, and disinfectants [18, 20–23]. These disinfectants are active against ASFV: Acids, alkalis, aldehydes, chlorines, and oxidizing agents [26]. From our experiments investigating the effect of pH on ASFV, it was concluded that low doses of acidic and alkaline disinfectants have extremely limited ability to disrupt the structure of the ASFV genome and inactivate the virus. Therefore, in this study, we selected several additional commercially available disinfectant products and tested their ability to inactivate ASFV. These included an RNase, RNA, and DNA remover, hydrogen peroxide (3%) (oxidant), alcohol (75%) (alcohol compound), and 84-disinfectant (with an effective chlorine content of 5%–7%). The disinfectant activity of chlorine compounds is based on the oxidation of peptide links, which denature proteins [45]. Oxidizing agents act as disinfectants by developing free hydroxyl radicals that oxidize lipids and nucleic acids [46, 47]. Alcohol (75%) absorbs water from the viral proteins, causing them to dehydrate, denature, and coagulate, damaging the viral envelope and easily inactivates the virus [17]. In this study, the ASFV genome was completely inactivated by disinfectants within 5 min, with only trace amounts of ASFV genome remaining, and it was completely undetectable after 30 min of treatment with disinfectants. Handwash and RNase, RNA and DNA remover, being the most effective disinfectants in this study, completely inactivated the virus genome within 5 min. In the laboratory, RNase, RNA, and DNA remover can be sprayed to remove ASFV; hand sanitizer is needed to ensure hand cleanliness during quarantine and control; and a large amount of alcohol or disinfectant should be sprayed on infected sites and trucks to ensure environmental safety.

## Conclusion

The ASFV genome remains stable in favorable environments but is also susceptible to standard disinfection methods. Standard disinfection methods are an effective way to inactive the ASFV genome. This study focused on the analysis of ASFV genome stability in different conditions and provided various standard disinfection methods for preventing and controlling ASF.

## Data Availability

The supplementary data can be available from the corresponding author up on a reasonable request.

## Authors’ Contributions

JX and YZ: Sample collection. JW and YC: Data collection. WZ, MC, and QZ: Laboratory work and data analysis. WZ and CT: Drafted the manuscript. CT and XZ: Study design and reviewed the manuscript. All authors have read, reviewed, and approved the final manuscript.
